# Introducing the Sudan Labor Market Panel Survey 2022

**DOI:** 10.4054/demres.2024.51.4

**Published:** 2024

**Authors:** Caroline Krafft, Ragui Assaad, Ruby Cheung

**Affiliations:** 1 St. Catherine University, Saint Paul, MN, USA.; 2 Humphrey School of Public Affairs, University of Minnesota, Minneapolis, MN, USA.; 3 St. Catherine University, Saint Paul, MN, USA.

## Abstract

**OBJECTIVE:**

This paper describes the new Sudan Labor Market Panel Survey (SLMPS) 2022, the first nationally representative survey in Sudan in almost a decade.

**METHODS:**

The paper details the design of the survey, including the topics covered by this multipurpose household survey and the complexities of the sampling strategy, which over-sampled refugees and the internally displaced. The training, fieldwork, resulting sample, and weights are described.

**CONTRIBUTION:**

The rich, publicly available data of the SLMPS provide substantial opportunities for researchers to better understand the evolution of Sudan’s labor market, economy, and society.

## 1. Introduction

This paper introduces the 2022 wave of the Sudan Labor Market Panel Survey (SLMPS 2022), the first wave of a planned, nationally representative longitudinal survey of labor market and broader socioeconomic conditions in Sudan. Sudan and other countries in the Middle East and North Africa region generally are a “data desert” and under-researched (Das et al. 2013; Ekhator-Mobayode and Hoogeveen 2021). The SLMPS 2022 is part of the Labor Market Panel Survey (LMPS) series conducted by the Economic Research Forum (ERF) since 1998. The LMPS series creates multipurpose household surveys that are publicly available for researchers. There are five LMPS waves to date completed in Egypt (1998, 2006, 2012, 2018, 2023^4^), two waves in Jordan (2010 and 2016), and one wave in Tunisia (2014) (Assaad et al. 2016; Krafft and Assaad 2021; Krafft, Assaad, and Rahman 2021; OAMDI 2016, 2018, 2019).

Prior to this, the most recent nationally representative survey data available for Sudan were from the 2014–2015 Sudan Household Budget Survey (SHBS) and the 2014 round of the Multiple Indicator Cluster Survey (MICS) (Central Bureau of Statistics [CBS] and UNICEF Sudan 2016). All nationally representative surveys previous to this, such as the Labor Force Survey or the Harmonized Household Health Survey, date to the period prior to the secession of South Sudan in 2011. The last population census was conducted in 2008.^5^ During COVID-19, mobile phone surveys were conducted,^6^ but mobile coverage is substantially less than universal in Sudan and mobile phone surveys were limited in length (Central Bureau of Statistics [CBS] and World Bank 2020; Krafft and Moylan 2023; Krafft, Nour, and Ebaidalla 2022; Osman, Rahasimbelonirina, and Etang 2022).

This paper describes the SLMPS 2022 data collection and data. Section 2 covers the household and individual questionnaire design and information on accessing the public use microdata. The complexities of the sample design are covered in Section 3, as well as the sample weights. Section 4 describes the processes of training and data collection. Section 5 presents a summary of key demographic and labor market statistics by sample strata. Section 6 concludes with a discussion of the strengths and limitations of this new data, as well as potential directions for future research.

## 2. Questionnaires and data availability

### 2.1 The questionnaires

The SLMPS questionnaires consist of a household questionnaire and an individual questionnaire, with modules detailed in Table 1. As there are 4,630 variables in the dataset, we present modules as a summary of the themes covered. Modules include not only labor market and economic topics but also demographic topics such as marriage, fertility, and migration, and social topics such as gender attitudes and time use. The modules built on and ensured substantial comparability with other LMPSs. ERF received a grant from the Data Production and Methods Unit of the Development Data Group at the World Bank to include specific modules and questions from the Living Standards Measurement Study Plus (LSMS+) surveys, which focus on gender-disaggregated asset, employment, and entrepreneurship data (World Bank 2023). The ERF research team worked closely with the LSMS+ team at the World Bank to incorporate these modules.

### 2.2 Public use microdata access

Public use microdata from the 2022 wave of the SLMPS are available from ERF’s Open Access Microdata Initiative (OAMDI) (OAMDI 2023). Researchers can request the microdata free of charge from https://www.erfdataportal.com/index.php/catalog/265. Documentation (questionnaires in English and Arabic, codebooks, and metadata) is also available through OAMDI. The other waves of LMPSs are also available from OAMDI, along with a harmonized dataset, with a subset of variables from all countries and waves, referred to as the Integrated Labor Market Panel Surveys.

## 3. Sampling and sample design

A fundamental challenge when designing the SLMPS sample was the lack of a recent, nationally representative sample frame. The last national population census in Sudan was in 2008, before the secession of South Sudan. There had also been limited updating of administrative borders and maps. The first level of administrative geography in Sudan is the state (*wilaya*), and there are 18 states in Sudan.^7^ The second level of administrative geography in Sudan is the locality (*mahaliya*), and CBS updated the borders of localities in 2017 to 189 distinct geographies (each locality nested within a single state). We used the updated borders combined with 2020 population estimates based on remote sensing data to create our sampling frame and draw our sample. We supplemented these sources with additional data to identify refugee and IDP camps and areas for our strata. We describe the details of this sampling frame and design below.

### 3.1 Summary of sample design: Planned and realized strata and primary sampling units

The planned sample design was a random stratified cluster sample made up of 5,000 households subdivided into 250 primary sampling units (PSUs). The strata represented in the sample are: (1) refugee camps, (2) refugee areas (areas with non-camp refugee settlements), (3) IDP camps, (4) IDP areas (areas with non-camp IDP settlements), (5) other (non-refugee/non-IDP) rural areas, and (6) other urban areas. We describe below the details of identifying these different geographies. These area strata were combined with states to create distinct state-area strata for our random stratified cluster sample. As discussed below, we created PSUs within each locality as rectangular geographic areas with similar populations as ascertained by the remote sensing data.

Table 2 shows the planned and realized sample by stratum and state. We successfully collected data from 250 PSUs as planned and from the various states as planned, with some deviations in the area types sampled as well as some replacement during fielding, as discussed below. In terms of the area types sampled, two fewer IDP camp PSUs than planned were sampled, one fewer in South Darfur and one fewer in East Darfur. (Other IDP camp PSUs were sampled in South Darfur but none in East Darfur. However, an additional IDP area PSU was sampled in East Darfur as the best replacement.)^8^ One fewer refugee area PSU than planned was sampled in South Darfur. (Other refugee area PSUs were sampled in South Darfur.) In both North Darfur and South Kordofan, one fewer IDP area PSU than planned was sampled, but in both cases, other IDP area PSUs were sampled in each governorate. The realized sample in a number of states added or subtracted one urban other and/or one rural other area, as these were often the backup PSUs. In all but one case, at least some of the planned urban and rural other areas were sampled. In West Darfur, the one planned rural other area was not sampled.^9^

### 3.2 Sample inputs

PSUs were created using publicly available datasets, QGIS, and Stata processing. The 2020 population estimates that underlie our sampling were created by WorldPop (WorldPop 2020). WorldPop created geoTIFF files with 100 × 100 meter (pixel) population estimates. Estimates are based on the official United Nations population estimates with dasymetric redistribution using random forest modeling and geospatial covariates (Stevens et al. 2015; WorldPop 2020). We used Sudan state and locality boundary files (OCHA Regional Office for Southern and Eastern Africa [ROSEA] 2020) to ensure that PSUs did not cross administrative boundaries.

We used two additional data sources to identify refugee camps/areas and IDP camps/areas. It is very unusual and a substantial contribution of the SLMPS to have a nationally representative sample of the displaced (Assaad, Krafft, and Wahba 2024). For refugees, we used geospatial data from UNHCR, updated in January 2021 (OCHA Sudan 2021). The dataset included points for a variety of location types, such as office, reception center, and crossing/entry point, as well as residential areas. We specifically used the locations for camps, refugee camps, collective self-settlement, open areas, and refugee settlement to identify our refugee camp and refugee area strata. For IDPs, we used round 1 of the Displacement Tracking Matrix (DTM) from the International Organization for Migration (IOM), from November 2020 (International Organization for Migration 2020). The data included points for a variety of location types, all of which were included as they contained households; they allowed us to distinguish camps from other types of IDP settlements.

### 3.3 Creating primary sampling units

**Figure 1:**
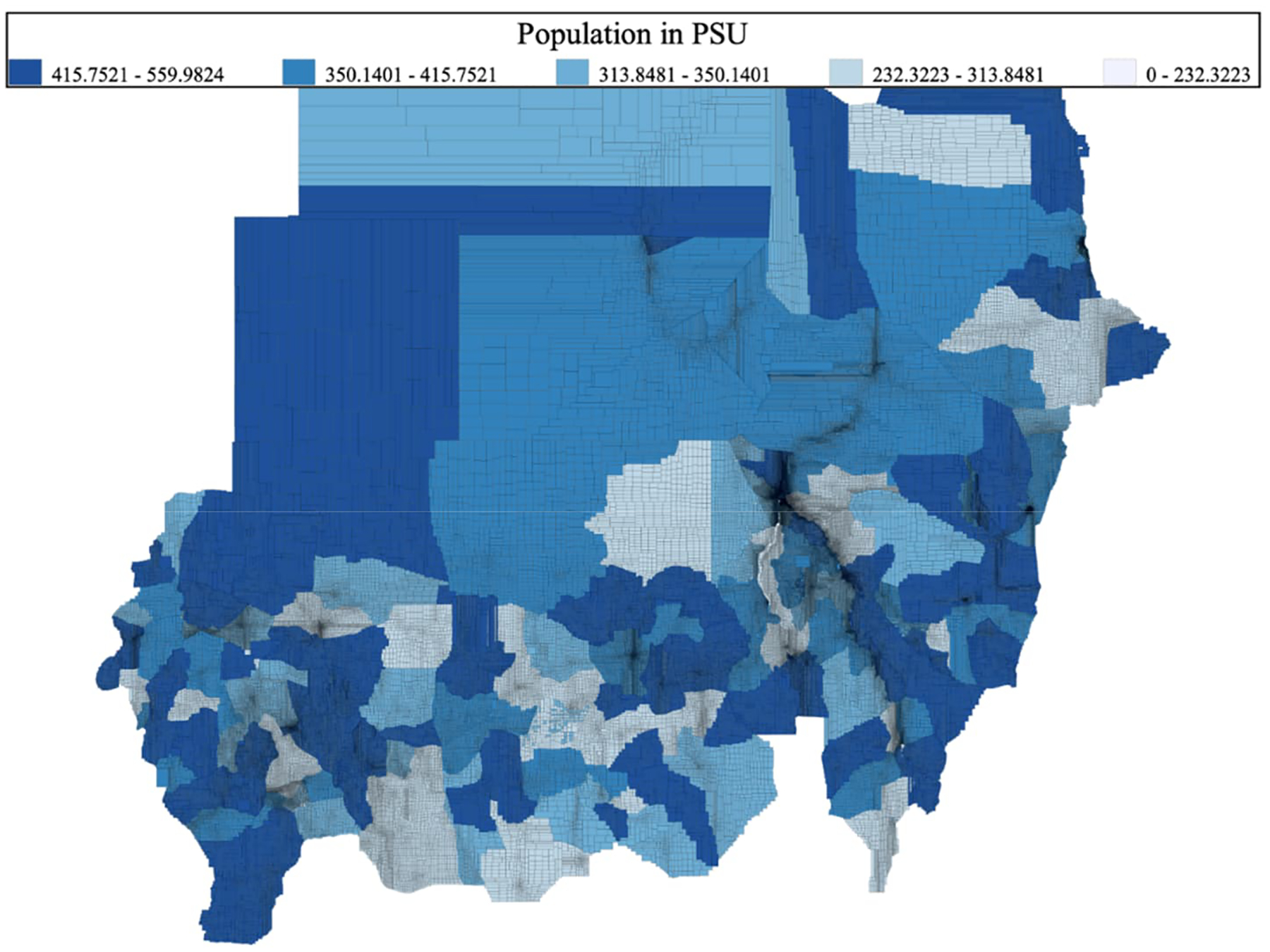
Full sample frame of PSUs, classified by individual population in the PSU *Source*: Authors’ construction based on WorldPop population estimates (WorldPop 2020) and locality boundaries (OCHA Regional Office for Southern and Eastern Africa [ROSEA] 2020). *Notes*: Black lines denote PSU borders. Areas with denser population may appear entirely black due to the borders.

We initially split the population pixels into separate files for each locality (using QGIS) to make a series of tractable datasets for creating PSUs. Looping over localities, we imported the geospatial data (shapefiles) into Stata.^10^ We set a maximum population for the PSU of 560 individuals, as we were aiming for a maximum size of 100 households per PSU for feasibility of listing in the field and the average household size in the 2008 census was 5.6 persons. We then undertook a splitting algorithm to generate tractable (rectangular) PSUs for fielding,^11^ splitting the locality (and subsequently subareas) in half (weighted by population, not by area) on latitude and then longitude and repeatedly splitting so long as the population was above the maximum threshold for each area. The resulting PSU areas had populations from 232 to 560 individuals and are illustrated in full for all of Sudan in Figure 1. Note that the areas that appear black (PSU borders are black) are those with high population densities.

**Figure 2:**
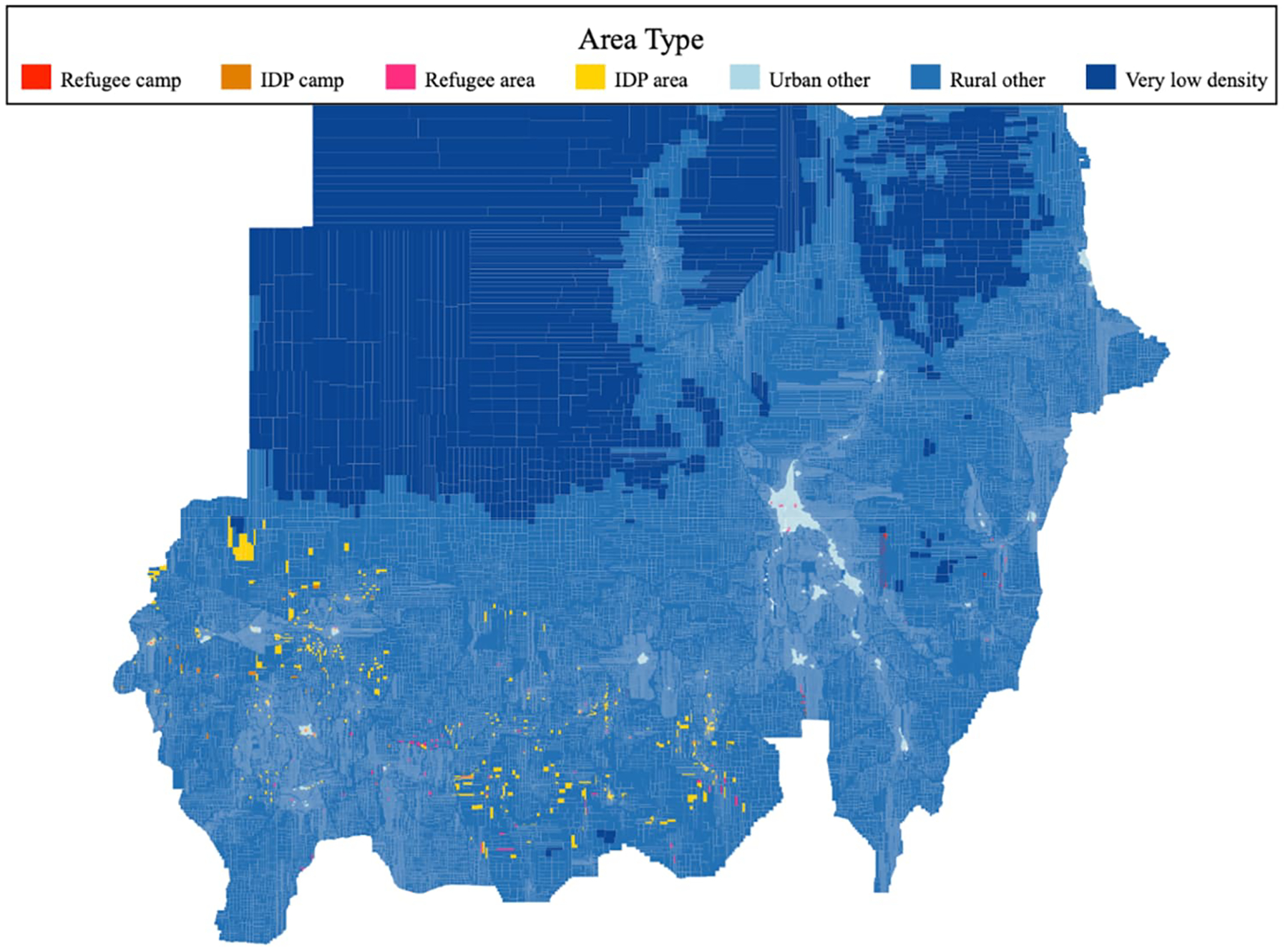
Full sample frame of PSUs, classified by area type *Source*: Authors’ construction based on WorldPop population projections (WorldPop 2020), locality boundaries (OCHA Regional Office for Southern and Eastern Africa [ROSEA] 2020), and data on locations of refugees (OCHA Sudan 2021) and IDPs (International Organization for Migration [IOM] 2020).

We then classified the PSUs into strata. We identified the PSUs containing the coordinates (single points) given for IDP and refugee camps^12^ and classified these as the IDP and refugee camp strata. We likewise identified the PSUs containing the coordinates for other (non-camp) types of IDP and refugee settlements, as well as PSUs whose centroid was within two kilometers^13^ of a refugee/IDP camp or a refugee/IDP settlement (but was not the location of a camp). We classified these as, variously, refugee or IDP areas. We then distinguished the urban or rural nature of other PSUs based on the density. Following UN Statistics guidance (United Nations Statistical Commission 2020) on measuring the degree of urbanization, we classified as urban any PSU where the population was at least 300 persons per square kilometer. Given the substantial amount of land in Sudan that is uninhabited and the practicalities of fieldwork, we also identified unpopulated areas based on a maximum density at the first percentile (fewer than 0.025 persons per square kilometer). We exclude this land area (which is estimated to have very limited population) from our sampling. The resulting sample frame consisted of 19 refugee camp PSUs, 63 IDP camp PSUs, 868 refugee area PSUs, 1,124 IDP area PSUs, 28,747 urban other PSUs, and 82,077 rural other PSUs, along with 1,149 excluded very low-density PSUs. The frame is mapped in Figure 2.

### 3.4 Sampling PSUs and households

Using this sample frame, we undertook a random stratified sample, with probability proportional to size (population) and without replacement in Stata,^14^ following the PSU sample sizes presented in Table 2. For fieldwork, we processed the resulting sample in QGIS and prepared it for listing of households on tablets using QField. With QField, fieldwork supervisors were instructed to canvas the entire PSU (based on the polygon boundaries mapped on their tablets) and place points for all the households in the area, numbering them sequentially (estimated per the sample frame to be 50–100 households). The number of households was then entered into the ODK-X (tablet data collection) supervisor program, which gave a random sample of 20 households from the number listed along with a random sample of up to 10 backups (in a random order). If there were 20 or fewer households in the field, all households were sampled. The 20 initial households could be fielded in whatever order was convenient, but if any ultimately were not part of the sample, the backups were to be used in the random order provided.

### 3.5 GPS coordinates

We received permission from CBS to make available in the public use version of the data GPS coordinates of the centroids of the sampled PSUs. To maintain confidentiality, we follow a displacement practice similar to the Demographic and Health Surveys and Living Standards Measurement Surveys (Burgert et al. 2013; Michler et al. 2022). From the true centroid of the PSU, we add a random displacement error of up to five kilometers in rural areas and up to two kilometers in urban areas. In an additional 1% of rural areas, the random displacement error was up to ten kilometers. We ensured that the displaced centroids remained in the true state and locality. The availability of these GPS coordinates will allow researchers to link the microdata of the SLMPS 2022 with readily available global geospatial data. For instance, the SLMPS 2022 data can be linked to land cover and weather condition data available at high geographic resolutions, thus opening up opportunities to study the impact of climate change and desertification on a wide variety of outcomes. It will also be possible to link the SLMPS 2022 data with geolocated data on conflict and civil unrest, allowing for the study of the impact of conflict on human populations.

### 3.6 Sample weights

From the sample design and data collected in the field, we can generate sample weights that are designed to make the SLMPS nationally representative. This section describes the creation of those weights. We start with the probability of a PSU being sampled. Denote a PSU as *p*. Denote a stratum as *s*. Denote a state as *t*. We sampled probability proportional to size based on the WorldPop 2020 population estimate (the sum of the population across the pixels within the PSU). Denote this PSU-specific population estimate as *E*_*p,t,s*_. We sampled a number of PSUs from each stratum-state (see realized cells in Table 2). Denote the number of PSUs completed as *C*_*t,s*_ and the total number of PSUs in the stratum-state as *P*_*t,s*_. The probability of a PSU being sampled from within a stratum-state is therefore:

(1) $$\pi_{p,t,s}=\frac{E_{p,t,s}*C_{t,s}}{\sum_{p=1}^{P_{t,s}}E_{p,t,s}}$$

That is, the population of the PSU, the number of clusters sampled in the stratum-state, and the total population across all PSUs (not just sampled PSUs) in the stratum-state determine the probability of a PSU being sampled. When the PSU population is higher, or more PSUs in the stratum-state are being sampled, the probability of being sampled is higher. When the total number of PSUs in the stratum-state is higher, the probability of a particular one being sampled is lower.

During fieldwork, the number of households within the PSU was supposed to be listed and recorded into ODK-X. In reality, in PSUs that turned out to have larger populations, supervisors did not fully list the PSU. After the fieldwork was completed, the CBS GIS team used satellite images to estimate the number of households within each PSU.^15^ Denote this listed population as *L*_*p,t,s*_. Denote the number of successfully completed households (this number accounts for non-responses) as *K*_*p,t,s*_. This number was supposed to be 20 per PSU but was smaller if the PSU turned out to be smaller than 20 households or if there was non-response. In some cases, more than 20 households per PSU were sampled to make up for shortfalls elsewhere. In all cases the weights account for these deviations. The probability of a particular household within a PSU being in the sample is thus:

(2) $$\rho_{p,t,s}=\frac{K_{p,t,s}}{L_{p,t,s}}$$

When a larger share of households in the PSU was completed, this probability is higher. It is lower for larger PSUs.

We combine the probability of the PSU being sampled and the probability of the household being sampled within the PSU to generate the PSU weight as the inverse probability of a particular household being sampled:

(3) $$w_{p,t,s}=\frac{1}{\pi_{p,t,s}*\rho_{p,t,s}}=\frac{1}{\frac{E_{p,t,s}*C_{t,s}}{\sum_{p=1}^{P_{t,s}}E_{p,t,s}}*\frac{K_{p,t,s}}{L_{p,t,s}}}=\frac{\left(\sum_{p=1}^{P_{t,s}}E_{p,t,s}\right)*L_{p,t,s}}{E_{p,t,s}*C_{t,s}*K_{p,t,s}}$$

Since, as discussed in the fieldwork description below, PSUs that appeared empty or to have fewer than five households were not fielded, these weights, unsurprisingly, sum to more than Sudan’s 2020 population. The weights also reflect substantial variability based on the listed populations of a finite number of PSUs. We therefore adjust weights to be equivalent to the sampling frame by stratum-state based on the 2020 population estimates,^16^ as follows:

(4) $${\tilde{w}}_{p,t,s}=\frac{w_{p,t,s}*\sum_{p=1}^{P_{t,s}}E_{p,t,s}}{\sum_{p=1}^{P_{t,s}}w_{p,t,s}}$$

This essentially multiplies all the weights within a stratum-state by a constant fraction based on the shares within each stratum-state in 2020.

A further complexity arose in terms of nationality and representation of non-Sudanese populations, particularly refugees, as well as Sudanese IDPs. Moreover, the national population presumably grew between 2020, the date of our sample frame, and mid-2022, when we fielded. We therefore adjust ${\tilde{w}}_{p,t,s}$ to reflect a 2022 mid-year national population of 46.9 million (United Nations Department of Economic and Social Affairs Population Division 2022). This population, *Q*, is divided into four groups, denoted as *g*. These groups are: refugee households (1.1 million individuals [UNHCR 2022]), non-refugee non-Sudanese households (250,000 individuals), IDP households (3.7 million individuals [UNHCR 2022]), and non-IDP Sudanese households (41.8 million). The adjustment for group *g* is:

(5) $${\tilde{w}}_{p,t,s,g}=\frac{{\tilde{w}}_{p,t,s}*I_{g}*Q_{g}}{\sum_{s=1}^{S}\sum_{t=1}^{T}\sum_{p=1}^{P_{t,s}}{\tilde{w}}_{p,t,s}*I_{g}}$$

where *I*_*g*_ is a dummy variable indicating that an individual is in group *g* and *Q*_*g*_ is the national population of group *g*. This modification adjusts the weights to reflect the population of different groups (IDPs, refugees, non-Sudanese, Sudanese) in 2022. These are the final household weights used in analyses.

### 3.7 Individual non-response and individual weights

Similar to visiting households up to three times in an attempt to collect data, interviewers visited individuals aged 5 and older up to three times to collect data from the individual him- or herself before a proxy respondent could be taken.^17^ Non-response occurred if an individual refused to respond or if on the third visit the individual was not available and a proxy was not available to respond on their behalf. Of the 21,057 individuals aged 5 and older in the household roster data, 20,086 (95%) consented to and completed the individual questionnaire. Among the 971 individuals who did not complete the individual questionnaire, 74 were not available on the third visit and no proxy was available. The remaining 897 refused; this number includes those under 18 who refused themselves or whose parents refused to consent for them to be interviewed. Individuals who did not respond were still among those listed by the household in the roster, so we have their basic demographic characteristics but not the detailed individual interview data.

We account for individual non-response and generate individual non-response weights, with covariates (*x*) for sex, age group, marital status, stratum, state, household nationality, household IDP status, and household refugee status. We interact sex with both age group and marital status as well. We estimate the predicted non-response rate as *r*_*x*_ based on a logit model.

We adjusted the household weight by this non-response to get the individual weight of:

(6) $$w_{p,t,s,g,x}=\frac{{\tilde{w}}_{p,t,s,g}}{1-r_{x}}$$

This weights up those individuals who did complete the individual questionnaire and have similar characteristics to those who were particularly likely not to respond.

## 4. Training and data collection

### 4.1 Planning and preparations

Planning for the SLMPS 2022 began as early as the fall of 2017, when ERF submitted a proposal to conduct the survey to the Growth and Labor Markets in Low Income Countries (GLM | LIC) research program of the Center for Labor Economics (IZA) in Bonn, Germany. While planning and preparations, such as adaptation work, had begun in fall 2018, protests against the Bashir regime in Sudan led to the regime’s eventual fall and a period of political instability. With the initiation of a transitional period marked by the establishment of a civilian-led government in partnership with the military in 2019, it was possible for ERF to resume efforts to conduct the survey in early 2020. Meetings were held in January 2020 with the Ministry of Finance, the Ministry of Labor and Social Development, and CBS, where in principle an agreement to implement the survey was reached. Several consultations with Sudanese researchers were held over the course of 2020 to adapt the LMPS questionnaire implemented in Egypt in 2018 to Sudanese conditions. A complete version of the questionnaire programmed for implementation on a tablet using ODK-X (Brunette et al. 2017) was available by November 2020.

After a series of delays associated with the onset of the COVID-19 pandemic and the turbulent economic situation in Sudan, including the lingering effects of sanctions on its banking system, a three-way contract involving what had then become the Ministry of Labor and Administrative Reform (MoLAR), CBS, and ERF was signed in April 2021. This led to the formation of a steering and technical committee involving various agencies of the Sudanese government, which would oversee implementation of the survey. A training of trainers (TOT) was conducted by ERF researchers and staff in May 2021 to train members of MoLAR and CBS on all aspects of implementing the survey. These trainers then led a two-week training camp organized by CBS for enumerators and supervisors in August 2021. The plan was to start the fieldwork in all 18 of Sudan’s states by September 2021.

A series of minor delays pushed the start of the fieldwork into October. Then the military-led coup against the civilian government happened on October 25, 2021, derailing the project timeline. ERF persisted and remained in constant contact with CBS over the ensuing months. As the political situation stabilized somewhat, it was decided to initiate fieldwork in May 2022 after a short online refresher training of the enumerators and supervisors. Fieldwork actually started on June 11, 2022, and most field operations were completed by the end of September 2022. The ERF research team received the anonymized raw data in November 2022, and the first beta version of the data was ready for analysis by January 2023, just a few months before the collapse of the Sudanese government and the breakout of hostilities on April 15, 2023.

### 4.2 Training

As mentioned above, a one-week TOT was organized by ERF for CBS and MoLAR staff in May 2021. The TOT included a review of the purpose of the survey, definitions of concepts, fielding procedures, and intensive practical training on administering the questionnaire on the tablet using previously written cases as well as mutual interviewing among trainees. The IT and statistics staff at CBS was also trained on data transmission, data handling, and data processing. A field pretest of the survey was carried out following the TOT on five PSUs not in the final sample and was used to make final modifications to the questionnaire.

In August 2021, a two-week training camp for enumerators and supervisors was organized in Aj-Jazira state. The trainees included 120 enumerators and 18 supervisors recruited locally in all 18 Sudanese states as well as a team of quality control enumerators recruited centrally. The trainers were made up of CBS and MoLAR staff who were trained in the TOT as well as one representative of the ERF research team. At the end of the training, a day of field practice was organized for the trainees to gain practical field interviewing skills.

Although fieldwork was supposed to start shortly after the completion of the enumerator training, a series of delays pushed it into October, and the coup that brought an end to the civilian-led government in Sudan happened on October 25, 2021. The ensuing political unrest forced a further postponement of the fieldwork. In April 2022, CBS informed ERF that conditions had stabilized sufficiently to relaunch the work. An online five-day refresher training for the enumerators and supervisors was organized in late May and early June 2022.

### 4.3 Fieldwork

Twenty enumerator teams were formed, one for each state except for Khartoum and South Darfur, which had two teams each. Teams varied in size from three to five enumerators and were led by one supervisor. Fieldwork began on June 11, 2022, and was completed by September 26, 2022.^18^ Due to the lack of a standard sampling frame, fieldwork proceeded in two steps, listing households and then surveying a random sample of listed households. First, supervisors were supposed to list all households within the borders of the PSU. The borders of the PSU were visualized using QField, a tablet-based geospatial mapping system that allowed the supervisors to mark down the GPS coordinates for each household within the PSU boundaries, give it a number, and take notes on its location.^19^ The supervisor then switched to the ODK-X application on the tablet and entered the total number of households. The tablet then provided a random sample of 20 households, which could be fielded in any order, and a backup random sample (to be fielded in the order given if one of the 20 households refused or that survey otherwise could not be completed).

Data collection for the randomly selected households was undertaken using the ODK-X survey program on tablets. Enumerators were instructed to attempt to visit a household up to three times, at least a day apart, before substituting a backup household. Likewise for individuals, up to three visits were to be conducted to attempt to have the individual respond for him- or herself. A proxy was used only if an individual was incapable of responding for him- or herself or on the third visit was still not available. Response by a proxy was marked in the questionnaire.

There were substantial electricity and internet connectivity challenges during the fieldwork stage. Power banks and solar chargers were used to recharge tablets. On tablets, ODK-X uses an SQLite database that allows for local storage of data even without an internet connection. When connectivity was available, data were exported to csv-formatted files and migrated to a Boxcryptor (encrypted) Dropbox folder for each enumerator on the tablet, allowing secure syncing as connectivity permitted with the central CBS office in Khartoum. The central office compiled the data across supervisors and enumerators.

### 4.4 Deviations from the planned sample in fielding implementation

The realities of the sample frame and the logistics of fielding led to a number of deviations from the planned sample in fielding. While the initial sample was *estimated* to have a reasonable number of households in each PSU based on satellite imaging and population projections, there were cases where a PSU did not in fact have any or many households. All PSU locations were reviewed first in the CBS offices to identify locations that were empty or where there appeared to be five or fewer households; these locations were replaced with backup PSUs. There were a variety of reasons why a PSU might have few or no households, including that it consisted of industrial/commercial (not residential) buildings, that it was a mine or grain storage area, or that it had rocks or grain silos that looked like residences.

When office review determined there were at least five potential households on the satellite maps, fielding was attempted. However, a number of issues arose in the field as well. During visits, some buildings were determined to be non-residential or abandoned. Furthermore, a number of locations were determined to be unsafe to field, a status that even changed and fluctuated frequently during the fieldwork. Persistent sandstorms also prevented fielding in specific localities. The rainy season likewise made some locations inaccessible for fielding.

Backup samples were created. Initially, one urban and one rural backup were provided per state, and further backups were drawn as needed to replace PSUs that could not be fielded. Backups were, if possible, from the same strata and always from the same state. When possible, additional backups were also drawn from the same locality in an attempt to minimize bias. However, there were cases when an entire locality became inaccessible.

Ultimately, 152 PSUs from the original sample of 250 were fielded in the initially planned locations. Nine of the initially planned backups were used. For the remainder, 24 were replaced by the first replacement given, 17 by the second, 17 by the third, 9 by the fourth, 6 by the fifth, 4 by the seventh, and the remaining 12 by various higher-order replacements. Repeated replacements tended to occur in localities with a high share of buildings that the population estimates likely mistook for residences (e.g., mines and grain storage areas).

The realized sample sizes also varied somewhat per PSU. (As discussed above, this is incorporated into the weights.) The smallest completed PSU had only 5 households and the largest had 29. The 25^th^ percentile was 19, the median 20, and from the median up through the 90th percentile was also 20. Smaller PSUs were generally the result of a PSU having fewer than 20 households. Refusals at the household level were relatively rare; only 81 households refused.

### 4.5 Quality control processes

Quality control took place throughout the fieldwork, along two main axes: quality control callbacks and desk review of data. For 5% of households, the CBS central office undertook callbacks targeting one random individual from within the household who had a mobile number. At the household level, half (51%) of households had a mobile, and 42% of individual questionnaire respondents aged 15+. Households and individuals were selected per enumerator to ensure that all enumerators were monitored. The entire household questionnaire was redone and the target individual’s questionnaire through the wages section (if applicable). A total of 275 quality control households were successfully completed.

Desk review of the data focused on ensuring data completion. ODK-X stores data in separate tables for different levels of data (household observation, individual observation, crop observation, parcel observation, etc.). These data are entered through linked subforms, which should be accessed through the parent household form to ensure data linkages. The data are also exported into separate tables as csv files. Although ODK-X can sync all tables with a server, given electrical and connectivity limitations, Boxcryptor and Dropbox with exports for each table were used. When households were finalized in the household forms, ODK-X checked that all data were present, including all linked forms. However, exporting, copying, and syncing were prone to human error, as well as errors in following the flow of forms. Desk review focused on ensuring that all observations at all levels were present, working with enumerators to re-export and sync when there were issues, and undertaking quality control calls and corrections when such issues could not be easily solved. The resulting data were cleaned to address a number of remaining issues (e.g., two enumerators entering the same household number).

## 5. Sample demographic and labor market characteristics

Table 3 presents SLMPS 2022 sample demographic and labor market characteristics by strata. In what follows we briefly discuss key patterns and differences across strata. Women are often slightly over-represented relative to men among strata, including the displaced (53%–54% of the sample in refugee camps, IDP camps, and IDP areas). IDP camp, IDP area, and rural other strata have particularly high shares of children aged 0–14 (46%–54% versus 38% in the urban other stratum). Around half (47%–55%) of those in IDP camp and IDP area strata identify as IDPs in the survey. In the refugee camp stratum, the share identifying as refugees in the survey is 94%; in the refugee area stratum it is 42%. Marital status (for ages 15+) varies across strata, with those in strata related to displacement often particularly likely to be widowed, including 10% of those in refugee camps. The share never married is lowest in the rural other stratum (28%), compared to 39% in the urban other stratum. There are enormous wealth disparities across strata, with the urban other stratum having the highest representation of households from the richest quintile (58%) and the refugee camp stratum having the highest representation from the poorest quintile (47%).

In terms of education and labor force outcomes, those aged 6–24 in the rural other stratum had the lowest enrollment rate (39%) compared to 73% in the urban other stratum. Correspondingly, the rural other stratum had the second-highest share of adults (aged 25–64) who were illiterate, 60%, surpassed by the refugee camp stratum (67%). The urban other stratum had the highest shares of its adult population with secondary (24%) or university (17%) educational attainment. Labor force participation rates (labor market outcomes are for ages 15–64) varied across strata, with IDP camp and IDP area strata having the lowest rates (33%) and urban other (40%) and refugee area strata (42%) the highest. Employment rates ranged from 31% to 39% and followed a pattern similar to that of labor force participation across strata. Unemployment rates as a share of the labor force were highest (14%) in refugee camp and urban other strata and lowest (5%–6%) in IDP camp and rural other strata.

## 6. Conclusions

The SLMPS 2022 data provide an updated, nationally representative sample for Sudan, where the most recent preceding survey was the SHBS 2014–2015 and the MICS 2014. The data allow for a substantial update on Sudan’s economy and society, albeit before conflict broke out in April 2023. ERF and CBS overcame a number of challenges to generate this publicly available data covering a wide variety of topics, with detailed household and individual components.

The SLMPS 2022 was carried out under very difficult economic and political conditions, which have undoubtedly affected the quality of the data collected. While some callbacks were carried out by enumerators at the headquarters of CBS to check on data quality, it was not possible to undertake field quality control of the state teams. The vast distances and poor travel conditions prevented the managers of the survey from carrying out many field visits or checking on the progress of the work. In some cases, supervisors and enumerators did not follow the instructions given to them. For instance, supervisors were instructed to enumerate all households located in a PSU irrespective of their number, but many stopped counting once they reached 100, necessitating the use of other, less reliable methods to ascertain the number of households in the PSU. In some instances, where PSUs contained both refugee camps and other non-camp populations, supervisors decided to enumerate only the non-camp populations. There were also many instances of a PSU being deemed too unsafe or infeasible to reach, necessitating substitutes be used. We attempted to address these deficiencies by adjusting the weights so that various population groups, such as refugees and IDPs, produce the known totals for these populations, but this does not mean that some biases have not crept into the data.

Despite all the challenges encountered in completing the SLMPS 2022, the data will serve as an invaluable resource as the only nationally representative socioeconomic data from Sudan in the turbulent period since 2014. Sudan’s economy and labor market have been substantially altered by the fall of the Bashir regime in 2019 and, following the SLMPS 2022, the conflagration of hostilities in April 2023. In fact, since the secession of South Sudan in 2011, the Sudanese economy has been in a state of perpetual crisis, which was only exacerbated by the political unrest that broke out in 2018.

Given the continuation of hostilities and the absence of an effective central government, data collection may not be feasible through 2024 and potentially longer. The SLMPS 2022 therefore provides an essential baseline of socioeconomic conditions in the country at a crucial time in its history. The nationally representative and large sample of IDPs and refugees will be particularly valuable for understanding the impacts of past conflicts in Sudan and potentially informing responses to the conflict that started in 2023. Given the longitudinal design of the survey, we hope to have an opportunity to revisit the same households when the political situation stabilizes to obtain an accurate update on their situations. We typically follow up LMPS samples at six-year intervals, and we hope that safety and stability are reestablished in Sudan well before any potential SLMPS 2028 wave. Furthermore, it may be possible to undertake brief phone surveys in the interim, at least for those with phones and retaining those numbers during the conflict, as CBS collected detailed contact information, including individual mobile numbers. This kind of data will not only be invaluable for relief and reconstruction efforts but also could facilitate research on the effect of conflict.

The SLMPS 2022 data provide unique insights into a number of issues given not only the large number of topics covered but also features such as the over-sampling of the displaced and GPS coordinates for each PSU, allowing for the linking of the SLMPS data with geospatial data. The brief summary statistics presented in this paper illustrate key challenges, such as low rates of labor force participation, employment, and school enrollment, as well as important disparities between urban and rural areas and by displacement status. Initial research using the SLMPS data to assess the welfare of the displaced (Assaad, Krafft, and Wahba 2024), labor markets and gender (Assaad, Jamkar, and Krafft 2024; Assaad, Krafft, and Wahby 2023; Krafft et al. 2023; Krafft and Moylan 2023), education (Ebaidalla and Rakhy 2024), and marriage and fertility (Sieverding 2024) is only scratching the surface of what is possible with the data. Now that the SLMPS 2022 data are publicly available, a wide variety of topics can be explored for Sudan.

## Figures and Tables

**Table 1:** Questionnaire modules

Household	Individual
Statistical Identification Individual Roster Housing Information Current Migration Remittances Other Income Shocks and Coping Nonagricultural Enterprises Characteristics Outside Employment Expenditures Assets Enterprise Revenue Agricultural Assets: Lands and Parcels Agricultural Assets: Livestock/Poultry Agricultural Assets: Capital Equipment Agricultural Crops Other Agricultural Income	Statistical Identification Residential Mobility Father’s Characteristics Mother’s Characteristics Siblings Health Education Training Skills Employment Unemployment Job Characteristics Secondary Job Labor Market History Marriage Fertility Female Employment Earnings Earnings in Secondary Job Return Migration (Sudanese) Immigration/Refugees (non-Sudanese) IDPs/Refugees (Sudanese) Information Technology Savings and Borrowing Attitudes Adult Time Use Child Time Use Rights: Parcels Rights: Livestock Rights: Durables

*Source*: Authors’ construction.

**Table 2:** Primary sampling units: Planned and realized, by state-area strata

Strata:	Refugee camp	IDP camp	Refugee area	IDP area	Urban other	Rural other	Total
State							
**Planned sample**							
Khartoum			17		15	2	34
North Darfur		3	1	8	4	1	17
South Darfur		5	3	7	1	1	17
West Darfur		3	2	3	1	1	10
East Darfur	1	1	1	1	6	4	14
Central Darfur		2	1	1		6	10
South Kordofan		1	1	5		3	10
Blue Nile					6	4	10
White Nile	6		1		5	2	14
Red Sea					8	5	13
Kassala	4		1		5	3	13
Gedaref	4		1		4	2	11
North Kordofan				1	10	6	17
Sannar					6	4	10
Aj Jazirah					10	7	17
River Nile					6	4	10
Northern					6	4	10
West Kordofan			1	4		8	13
**Total**	15	15	30	30	93	67	250
**Realized sample**							
Khartoum			17		15	2	34
North Darfur		3	1	7	5	1	17
South Darfur		4	2	7	2	2	17
West Darfur		3	2	3	2		10
East Darfur	1		1	2	7	3	14
Central Darfur		2	1	1		6	10
South Kordofan		1	1	4		4	10
Blue Nile					7	3	10
White Nile	6		1		5	2	14
Red Sea					9	4	13
Kassala	4		1		6	2	13
Gedaref	4		1		4	2	11
North Kordofan				1	9	7	17
Sannar					7	3	10
Aj Jazirah					10	7	17
River Nile					5	5	10
Northern					6	4	10
West Kordofan			1	4		8	13
**Total**	15	13	29	29	99	65	250

*Source*: Authors’ construction based on planned sample and SLMPS 2022 realized sample.

**Table 3:** Sample demographic and labor market characteristics (percentages), by strata

	Refugee camp	IDP camp	Refugee area	IDP area	Urban other	Rural other	Total
**Sex**							
Male	46.4	46.9	50.5	46.0	48.4	50.3	49.8
Female	53.6	53.1	49.5	54.0	51.6	49.7	50.2
**Age group**							
0–4	14.6	22.5	12.7	17.1	14.6	20.4	18.8
5–9	13.0	16.5	12.9	14.2	12.1	13.5	13.1
10–14	16.0	14.9	16.0	15.1	11.2	13.2	12.7
15–19	11.4	8.5	14.0	10.8	10.1	9.4	9.6
20–24	9.3	7.7	8.5	8.3	9.3	7.9	8.3
25–29	4.9	6.8	6.4	5.4	7.0	7.0	7.0
30–34	3.9	5.2	5.4	4.4	6.2	5.9	5.9
35–39	5.0	5.0	4.9	4.8	5.9	4.5	4.9
40–44	5.3	4.3	4.8	3.9	4.4	4.3	4.4
45–49	4.2	2.5	3.4	4.7	4.4	3.0	3.4
50–54	2.7	2.1	3.7	3.2	4.1	2.9	3.2
55–59	1.5	0.6	1.3	2.7	2.8	1.5	1.9
60–64	2.9	1.0	2.1	1.5	2.9	2.5	2.6
65–69	1.7	0.4	1.0	1.3	1.9	1.5	1.6
70–74	1.6	0.9	1.8	0.8	1.8	1.2	1.3
75+	2.0	1.1	1.0	1.9	1.4	1.5	1.4
**IDP**	1.9	55.2	15.0	47.1	7.3	7.3	7.9
**Refugee**	94.4	0.0	41.6	0.0	6.8	0.0	2.4
**Marital status (ages 15+)**							
Never married	35.0	31.1	45.7	33.4	38.7	27.6	31.2
Contractually married	0.0	0.5	0.3	1.6	0.3	0.3	0.3
Married	50.3	60.4	45.9	54.2	53.7	66.7	62.4
Divorced	5.1	4.1	3.2	4.6	2.4	2.0	2.2
Widowed	9.6	3.9	4.9	6.2	4.9	3.4	3.9
**Quintiles of household wealth (percentage of households)**							
Poorest	46.8	26.7	14.6	15.4	3.1	28.7	22.0
Poorer	22.8	19.0	12.6	27.6	2.6	24.0	18.5
Middle	16.4	36.1	12.5	31.7	7.2	23.8	19.6
Richer	12.0	16.8	36.3	22.7	29.3	16.3	19.9
Richest	2.1	1.4	24.0	2.5	57.8	7.1	20.0
**Student (enrolled, ages 6–24)**	60.6	63.5	75.9	63.9	73.3	39.3	48.9
**Educational attainment (ages 25–64)**							
Illiterate	66.9	44.0	36.3	51.2	23.2	60.3	49.0
Reads and writes	14.3	19.2	13.2	17.1	12.7	15.7	14.8
Primary	9.6	17.8	22.7	12.2	19.7	11.1	13.8
Secondary	7.4	13.1	19.7	12.1	24.4	8.1	13.1
Post-secondary	0.1	0.4	0.8	0.7	1.8	0.3	0.8
University	1.7	5.5	7.1	6.6	16.5	4.3	7.9
Post-graduate	0.0	0.0	0.3	0.1	1.7	0.1	0.6
**Labor force participation rate (% of population aged 15–64)**	36.0	32.6	42.1	33.1	40.3	36.9	37.9
**Employed (% of population aged 15–64)**	30.9	31.0	38.8	30.8	34.7	34.8	34.8
**Unemployment rate (% of labor force aged 15–64)**	14.1	4.9	7.8	7.0	13.9	5.9	8.4
**N (individual observations in strata)**	1,392	1,302	3,227	3,018	10,152	6,351	25,442

*Source*: Authors’ calculations based on SLMPS 2022.

## Footnotes

4 The Egyptian LMPS 2023 data will be publicly available in October 2024.

5 In Krafft, Assaad, and Cheung (2023) we validate and compare the SLMPS 2022 data against these other data sources. Key features of the labor market, such as high rates of self-employment and the primacy of agricultural income, are also similar when comparing COVID-19 high-frequency surveys and the SLMPS 2022 (Assaad, Jamkar, and Krafft 2024; Krafft, Nour, and Ebaidalla 2022; Osman, Rahasimbelonirina, and Etang 2022).

6 Seven waves of World Bank high-frequency COVID-19 phone surveys, covering 2020–2022, are publicly available at https://microdata.worldbank.org/index.php/catalog/4552/. Two waves of International Labour Organization–sponsored and ERF-implemented COVID-19 phone surveys are publicly available at https://www.erfdataportal.com/index.php/catalog/230.

7 The Abyei area, which under current peace terms is part of both Sudan and South Sudan, was combined with West Kordofan for sampling purposes.

8 We therefore combine the IDP camp and IDP area populations in East Darfur when weighting.

9 We therefore combine the rural other populations of West Darfur and neighboring Central Darfur when weighting.

10 Using the shp2dta command.

11 Boundaries were modified in QGIS to respect locality borders and fix any overlaps, making a few PSUs nonrectangular in fielding.

12 Using the Stata command geoinpoly.

13 Using the Stata command geonear.

14 Using the Stata command gsample.

15 Calculations were based on the ratio of buildings observed on the map to the number listed in the field for the fraction of the PSU the supervisor did list. This ratio and the number of buildings in the listed area were used to generalize to the PSU as a whole based on the fraction of the PSU the listed area covered.

16 Only national estimates are available for 2022, not state- and stratum-specific estimates.

17 Parents of children 5 to 17 had the option to respond on their children’s behalf at any time.

18 A few interviews were reviewed and revised over the phone thereafter.

19 A household was considered within the PSU if the front door/entrance of the dwelling was within the boundaries.
